# Effect of Remimazolam on Postoperative Delirium in Surgical ICU Patients: A Single‐Center Prospective Cohort Study

**DOI:** 10.1155/ccrp/3315388

**Published:** 2026-06-18

**Authors:** Jiajia Hong, Chengjin Zhao, Lin Lin

**Affiliations:** ^1^ Department of Critical Care Medicine, The First Affiliated Hospital, Sun Yat-sen University, Guangzhou, Guangdong, China, sysu.edu.cn; ^2^ Guangdong Clinical Research Center for Critical Care Medicine, The First Affiliated Hospital, Sun Yat-sen University, Guangzhou, Guangdong, China, sysu.edu.cn; ^3^ Department of Critical Care Medicine, Ningming Branch of The Affiliated Minzu Hospital of Guangxi Medical University, Guangxi Zhuang Autonomous Region, Chongzuo, China

**Keywords:** inverse probability of treatment weighting, mechanical ventilation, postoperative delirium, remimazolam, sedation, surgical intensive care unit

## Abstract

**Aim:**

Postoperative delirium is a clinically important complication among patients admitted to a surgical intensive care unit (ICU). Remimazolam is increasingly used for perioperative and ICU sedation, but its association with postoperative delirium after elective surgery remains uncertain. This study evaluated the association between remimazolam‐based postoperative sedation and postoperative delirium in surgical ICU patients.

**Design:**

A single‐center prospective cohort study.

**Methods:**

Adult patients admitted to a surgical ICU after elective surgery between June 2024 and May 2025 were categorized according to their early postoperative sedation strategy. The remimazolam group was compared with a nonremimazolam sedation strategy group, which included patients receiving dexmedetomidine, midazolam, propofol/ciprofol, or no continuous sedative infusion. The primary outcome was postoperative delirium within the first 5 postoperative days or during the ICU stay, whichever was shorter. Delirium was assessed using the Confusion Assessment Method for the ICU and/or the Intensive Care Delirium Screening Checklist when patients were sufficiently arousable. Stabilized inverse probability of treatment weighting was used to reduce measured baseline imbalance. Weighted logistic regression was used for delirium, and weighted median regression was used for skewed continuous outcomes.

**Results:**

Of 307 patients assessed for eligibility, 208 were enrolled, and 204 were included in the final analysis, of whom 125 received remimazolam and 79 received nonremimazolam sedation strategies. Postoperative delirium occurred in 15 patients (12.0%) in the remimazolam group and 7 patients (8.9%) in the comparator group. After inverse probability weighting, remimazolam was not statistically significantly associated with postoperative delirium (adjusted odds ratio 1.69, 95% confidence interval 0.61–4.69; *p* = 0.312). Mechanical ventilation duration was longer in the remimazolam group (median difference 6.00 h, 95% confidence interval 2.00–8.00; *p* < 0.001).

**Conclusion:**

Remimazolam was not statistically significantly associated with postoperative delirium compared with nonremimazolam sedation strategies. However, the low event rate, wide confidence interval, and heterogeneous comparator group mean that clinically important differences cannot be excluded. The longer ventilation duration observed in the remimazolam group should be interpreted cautiously because residual confounding may remain.

**Trial Registration:** Chinese Clinical Trial Registry: ChiCTR2400085877

## 1. Background

Postoperative delirium is an acute and fluctuating disturbance of attention, awareness, and cognition that occurs after major surgery and during critical illness. It is associated with prolonged hospitalization, increased healthcare costs, functional decline, long‐term cognitive impairment, and increased mortality [1–4].

Sedation practice is one of the potentially modifiable factors related to delirium risk. Contemporary ICU guidelines generally favor light sedation and recommend avoiding benzodiazepine‐based sedation when feasible, partly because traditional benzodiazepines have been associated with longer ventilation and delirium in critically ill patients [5, 6]. Dexmedetomidine has also been studied as a light sedation strategy in critically ill patients, although its effects may vary by population and clinical context [7].

Remimazolam is an ultra‐short‐acting benzodiazepine with rapid onset and offset, metabolism through tissue esterases, and reversibility with flumazenil. These properties make it attractive for procedural sedation, anesthesia, and potentially ICU sedation. Recent randomized studies in mechanically ventilated ICU patients have reported effective short‐term sedation and favorable hemodynamic profiles compared with propofol or dexmedetomidine [8–13]. However, these studies mainly focused on sedative efficacy, hemodynamic safety, or short‐term procedural outcomes. Evidence regarding postoperative delirium remains limited, especially in real‐world surgical ICU practice.

To address this evidence gap, we conducted a single‐center prospective cohort study to evaluate whether remimazolam‐based postoperative sedation was associated with postoperative delirium in adult surgical ICU patients after elective surgery.

## 2. Methods

### 2.1. Study Design, Setting, and Participants

This single‐center prospective cohort study was conducted in the surgical ICU of the First Affiliated Hospital of Sun Yat‐sen University from June 2024 to May 2025. The study followed the principles of the Strengthening the Reporting of Observational Studies in Epidemiology (STROBE) statement [14].

Eligible patients were adults admitted to the surgical ICU after elective surgery and considered by the treating ICU team to require postoperative observation and management. The surgical population included patients undergoing cardiovascular surgery, thoracic surgery, major vascular surgery, and otolaryngologic head and neck surgery. Patients were excluded if they had preoperative cognitive dysfunction or altered mental status, alcohol withdrawal, postoperative coma or seizures, planned repeat elective surgery during the same hospitalization, or incomplete key outcome data.

### 2.2. Exposure Definition and Sedation Strategies

The exposure of interest was the initial postoperative sedation strategy during the early ICU period. Patients were categorized into the remimazolam group if remimazolam was used as the primary sedative strategy after ICU admission. Patients were categorized into the nonremimazolam sedation strategy group if they received dexmedetomidine, midazolam, propofol/ciprofol, or no continuous sedative infusion according to the attending physician’s decision. The comparator group therefore reflected pragmatic real‐world postoperative sedation practice rather than a single standardized sedative regimen.

Sedation and analgesia were titrated according to local practice and the clinical condition of the patient, with the aim of maintaining light‐to‐moderate sedation when clinically appropriate. Sedation depth, ventilator synchrony, hemodynamic status, pain control, and extubation readiness were assessed routinely by the ICU team. Sedative and analgesic agents were reduced or discontinued when patients were considered ready for awakening and extubation.

### 2.3. Delirium Assessment

The primary outcome was postoperative delirium within the first 5 postoperative days or during the ICU stay, whichever was shorter. Delirium was assessed using the Confusion Assessment Method for the ICU (CAM‐ICU) and/or the Intensive Care Delirium Screening Checklist (ICDSC) by trained ICU clinicians or nurses during routine postoperative care. These instruments are widely used for delirium screening in critically ill and postoperative patients [15, 16]. Assessments were performed when patients were sufficiently arousable for evaluation, including during light sedation, sedation interruption, and after sedative discontinuation. Assessments were not considered valid during deep sedation when the patient could not be evaluated reliably.

### 2.4. Outcomes

The primary outcome was postoperative delirium. Secondary outcomes included duration of mechanical ventilation, ICU length of stay, hospital length of stay, C‐reactive protein (CRP) level on postoperative day 2, and relative CRP change from postoperative day 1 to postoperative day 2. Duration of mechanical ventilation was taken from the main study database, as prespecified for this revision.

### 2.5. Data Collection

Baseline and perioperative variables were prospectively collected, including sex, age, body mass index, educational level, hypertension, diabetes, alcohol consumption, smoking, surgery type, American Society of Anesthesiologists classification, surgery duration, estimated blood loss, use of cardiopulmonary bypass, APACHE II score, and postoperative CRP levels. Missing data were limited and were handled as described below.

### 2.6. Data Analysis

Continuous variables are presented as medians with interquartile ranges, and categorical variables are presented as counts and percentages. Between‐group comparisons before weighting were performed using the Mann–Whitney *U* test for continuous variables and the chi‐square or Fisher exact test for categorical variables, as appropriate. A two‐sided *p* value < 0.05 was considered statistically significant.

To reduce measured confounding, stabilized IPTW was applied. The propensity score model included sex, age, APACHE II score, educational level, body mass index, hypertension, diabetes, alcohol consumption, smoking, surgery type, American Society of Anesthesiologists classification, surgery duration, blood loss, CRP on postoperative day 1, and systemic circulation use. Covariate balance was assessed using standardized mean differences, with values below 0.1 considered acceptable [17].

Weighted logistic regression was used to estimate the association between remimazolam and postoperative delirium. Weighted quantile regression with tau = 0.5 was used for skewed continuous outcomes, and results are reported as median differences with 95% confidence intervals [18]. The 95% confidence intervals for median differences were estimated using nonparametric bootstrap resampling with 1000 repetitions. Odds ratios were obtained by exponentiating logistic regression coefficients.

Sensitivity analyses were performed to address heterogeneity in the comparator group. The nonremimazolam group was stratified into no continuous sedative infusion, dexmedetomidine, midazolam, and propofol/ciprofol. Firth penalized logistic regression was used for subgroup comparisons of delirium to reduce small‐sample bias and handle zero‐event cells [19]. For continuous outcomes, weighted median regression was repeated in subgroup analyses and after excluding zero‐event subgroups.

### 2.7. Ethical Considerations

Ethics approval was obtained from the Ethics Committee of the First Affiliated Hospital of Sun Yat‐sen University (approval number: NO. 2023‐899). The study was registered in the Chinese Clinical Trial Registry. Written informed consent was obtained from all participants or their legally authorized representatives before enrollment.

## 3. Results

### 3.1. Patient Characteristics and Comparator Strategies

**FIGURE 1:**
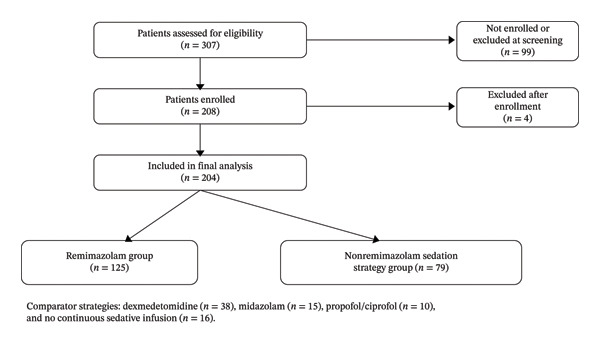
Study flow diagram.

During the study period, 307 consecutive patients were assessed for eligibility. Of these, 99 were not enrolled or were excluded during screening, and 208 patients were enrolled. Four enrolled patients were subsequently excluded according to the predefined exclusion criteria. A total of 204 verified individual patient records were included in the final analysis: 125 in the remimazolam group and 79 in the non‐remimazolam sedation strategy group. The comparator group included 38 patients receiving dexmedetomidine, 15 receiving midazolam, 10 receiving propofol/ciprofol, and 16 receiving no continuous sedative infusion. The patient screening and enrollment process is shown in Figure 1.

**FIGURE 2:**
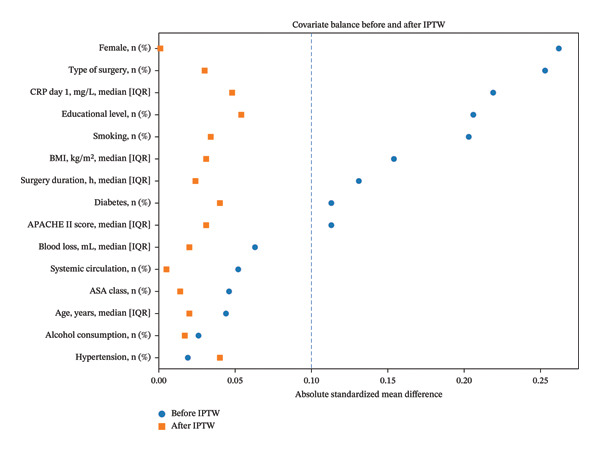
Covariate balance before and after inverse probability of treatment weighting. The dashed vertical line indicates standardized mean difference = 0.1. IPTW, inverse probability of treatment weighting; SMD, standardized mean difference.

Before IPTW adjustment, several covariates showed imbalance between groups, including sex, body mass index, educational level, CRP on postoperative day 1, surgery duration, surgery type, and APACHE II score. After IPTW adjustment, measured baseline covariates were well balanced, with standardized mean differences below 0.1 for all variables (Table 1 and Figure 2).

**TABLE 1:** Baseline characteristics before and after inverse probability of treatment weighting.

Characteristic	Overall	Remimazolam before	Control before	*p* before	SMD before	Remimazolam after IPTW	Control after IPTW	*p* after	SMD after
Female, *n* (%)	77 (37.7)	41 (32.8)	36 (45.6)	0.076	0.262	47.7 (37.8)	29.0 (37.8)	0.997	0.001
Age, years, median [IQR]	55.5 [43.0, 64.2]	55.0 [43.0, 64.0]	56.0 [45.5, 65.0]	0.536	0.044	56.0 [43.0, 64.4]	55.2 [44.2, 64.8]	0.890	0.020
BMI, kg/m^2^, median [IQR]	24.0 [22.0, 26.0]	24.0 [22.0, 26.5]	23.0 [21.8, 25.9]	0.312	0.154	24.0 [21.5, 26.0]	24.0 [22.0, 26.0]	0.833	0.031
Educational level, *n* (%)				0.351	0.206				0.054
High	44 (21.6)	30 (24.0)	14 (17.7)			26.6 (21.0)	15.5 (20.1)		
Low	52 (25.5)	30 (24.0)	22 (27.8)			32.6 (25.8)	19.6 (25.5)		
Medium	101 (49.5)	59 (47.2)	42 (53.2)			62.9 (49.8)	39.8 (51.9)		
Unknown	7 (3.4)	6 (4.8)	1 (1.3)			4.3 (3.4)	1.9 (2.5)		
Hypertension, *n* (%)	66 (32.4)	40 (32.0)	26 (32.9)	1.000	0.019	42.9 (34.0)	27.6 (35.9)	0.783	0.040
Diabetes, *n* (%)	31 (15.2)	17 (13.6)	14 (17.7)	0.431	0.113	19.7 (15.6)	10.9 (14.2)	0.784	0.040
Alcohol consumption, *n* (%)	40 (19.6)	24 (19.2)	16 (20.3)	0.858	0.026	24.3 (19.2)	14.2 (18.5)	0.905	0.017
Smoking, *n* (%)	55 (27.0)	38 (30.4)	17 (21.5)	0.196	0.203	32.4 (25.7)	18.6 (24.2)	0.814	0.034
CRP Day 1, mg/L, median [IQR]	2.8 [1.4, 6.7]	3.0 [1.3, 6.6]	2.7 [1.5, 7.2]	0.851	0.219	3.1 [1.3, 7.0]	2.7 [1.5, 6.0]	0.735	0.048
Surgery duration, h, median [IQR]	6.0 [4.2, 7.0]	6.0 [4.2, 7.1]	5.4 [4.0, 7.0]	0.395	0.131	5.6 [4.2, 7.0]	5.9 [4.0, 7.5]	0.866	0.024
Blood loss, mL, median [IQR]	500.0 [300.0, 800.0]	500.0 [300.0, 800.0]	500.0 [300.0, 800.0]	0.411	0.063	500.0 [300.0, 800.0]	500.0 [215.5, 637.2]	0.892	0.020
Systemic circulation, *n* (%)	145 (71.1)	90 (72.0)	55 (69.6)	0.753	0.052	87.6 (69.4)	53.5 (69.6)	0.973	0.005
Type of surgery, *n* (%)				0.184	0.253				0.030
Cardiac	165 (80.9)	105 (84.0)	60 (75.9)			99.8 (79.0)	60.3 (78.5)		
ENT	21 (10.3)	9 (7.2)	12 (15.2)			15.3 (12.1)	9.0 (11.8)		
Other	18 (8.8)	11 (8.8)	7 (8.9)			11.2 (8.9)	7.5 (9.7)		
ASA class, *n* (%)				0.969	0.046				0.014
2	24 (11.8)	14 (11.2)	10 (12.7)			15.9 (12.6)	9.8 (12.8)		
3	156 (76.5)	96 (76.8)	60 (75.9)			96.3 (76.2)	58.1 (75.6)		
4	22 (10.8)	14 (11.2)	8 (10.1)			13.1 (10.4)	8.2 (10.7)		
Unknown	2 (1.0)	1 (0.8)	1 (1.3)			1.1 (0.8)	0.7 (0.9)		
APACHE II score, median [IQR]	10.0 [7.0, 13.0]	10.0 [7.0, 14.0]	10.0 [6.5, 13.0]	0.558	0.113	9.0 [6.0, 13.0]	10.0 [6.0, 13.0]	0.832	0.031

*Note:* Values are *n* (%) or median [interquartile range] unless otherwise indicated. Weighted rows are presented as weighted counts (%) or weighted medians [interquartile range].

Abbreviations: APACHE II, acute physiology and chronic health evaluation II; CRP, C‐reactive protein; ICU, intensive care unit; IPTW, inverse probability of treatment weighting; SMD, standardized mean difference.

### 3.2. Primary Outcome

Postoperative delirium occurred in 22 patients (10.8%) in the overall cohort. In the unweighted analysis, delirium occurred in 15 of 125 patients (12.0%) in the remimazolam group and 7 of 79 patients (8.9%) in the nonremimazolam group (*p* = 0.644). After IPTW adjustment, remimazolam was not statistically significantly associated with postoperative delirium (adjusted odds ratio 1.69, 95% confidence interval 0.61–4.69; *p* = 0.312) (Tables 2 and 3).

**TABLE 2:** Unweighted primary and secondary outcomes.

Outcome	Overall (*n* = 204)	Remimazolam (*n* = 125)	Nonremimazolam (*n* = 79)	*p*
Delirium, *n* (%)	22 (10.8)	15 (12.0)	7 (8.9)	0.644
Mechanical ventilation duration, h, median [IQR]	8.0 [5.0, 16.2]	12.0 [6.0, 17.0]	6.0 [5.0, 10.5]	< 0.001
ICU length of stay, h, median [IQR]	21.0 [18.3, 39.9]	21.5 [18.5, 40.2]	20.9 [18.1, 34.7]	0.303
Hospital length of stay, d, median [IQR]	11.0 [8.0, 15.0]	11.5 [7.0, 16.0]	11.0 [8.0, 14.5]	0.840
CRP Day 2, mg/L, median [IQR]	55.97 [32.59, 74.24]	52.39 [32.40, 68.07]	59.63 [38.96, 77.66]	0.106
Relative CRP change, %, median [IQR]	1285.0 [478.7, 3666.9]	1224.7 [481.1, 3651.0]	1435.4 [475.0, 3585.6]	0.731

*Note:* Values are *n* (%) or median [interquartile range].

Abbreviations: CRP, C‐reactive protein; ICU, intensive care unit.

**TABLE 3:** Adjusted effect estimates after stabilized inverse probability of treatment weighting.

Outcome	Model	OR or median difference (95% CI)	*p*
Delirium, *n* (%)	Weighted logistic regression	1.69 (0.61, 4.69)	0.312
Duration of mechanical ventilation, h	Weighted median regression (*τ* = 0.5)	6.00 (2.00, 8.00)	< 0.001
ICU length of stay, h	Weighted median regression (*τ* = 0.5)	0.48 (−0.86, 4.19)	0.500
Hospital length of stay, d	Weighted median regression (*τ* = 0.5)	1.00 (−2.00, 2.43)	1.000
Relative change in CRP, %	Weighted median regression (*τ* = 0.5)	−203.60 (−1088.26, 245.30)	0.320

*Note:* Weighted logistic regression was used for delirium. Weighted median regression (tau = 0.5) was used for continuous outcomes.

Abbreviations: CI, confidence interval; CRP, C‐reactive protein; ICU, intensive care unit; OR, odds ratio.

### 3.3. Secondary Outcomes

The duration of mechanical ventilation was longer in the remimazolam group than in the comparator group. Before weighting, median ventilation duration was 12.0 h in the remimazolam group and 6.0 h in the nonremimazolam group (*p* < 0.001). After IPTW adjustment, remimazolam remained associated with longer mechanical ventilation duration (adjusted median difference 6.00 h, 95% confidence interval 2.00–8.00; *p* < 0.001).

No statistically significant adjusted differences were observed for ICU length of stay, hospital length of stay, or relative CRP change. The adjusted median difference was 0.48 h for ICU length of stay (*p* = 0.500), 1.00 day for hospital length of stay (*p* = 1.000), and −203.60% for relative CRP change (*p* = 0.320) (Table 3).

### 3.4. Sensitivity Analyses

Sensitivity analyses stratifying the comparator group by sedation strategy were exploratory because of small subgroup sizes and zero‐event cells. In multigroup comparisons, estimates for delirium were imprecise and did not show statistically significant differences between remimazolam and any specific comparator subgroup. For mechanical ventilation duration, the direction and magnitude of estimates varied across comparator strategies. These results should be interpreted as descriptive and hypothesis‐generating rather than confirmatory (Supporting Tables S1 and S2).

## 4. Discussion

In this prospective cohort study of postoperative surgical ICU patients, remimazolam was not statistically significantly associated with postoperative delirium compared with nonremimazolam sedation strategies after IPTW adjustment. The point estimate, however, was above 1, and the confidence interval was wide. This result is therefore better read as inconclusive rather than reassuring. Remimazolam was also associated with a longer duration of mechanical ventilation, while ICU length of stay, hospital length of stay, and CRP change did not differ clearly between groups.

The low number of delirium events is central to the interpretation of this study. Postoperative delirium occurred in 22 patients overall. With such a small number of events, the analysis had limited power to detect moderate differences between groups. A nonsignificant *p* value in this setting does not establish equivalence and does not exclude clinically important harm. This point is particularly relevant because the confidence interval around the adjusted odds ratio remained broad.

The comparator group also needs careful consideration. In daily ICU practice, sedative choice is shaped by hemodynamics, expected ventilation time, surgical complexity, and the clinician’s judgment at the bedside. The nonremimazolam group in this study therefore included dexmedetomidine, midazolam, propofol or ciprofol, and patients without continuous sedative infusion. These strategies are not pharmacologically interchangeable. Pooling them provides a pragmatic comparison with routine practice, but it weakens interpretation as a drug‐specific comparison. For this reason, the subgroup analyses were treated as exploratory only.

Recent randomized studies have provided useful information on remimazolam for ICU sedation. A randomized trial in postoperative mechanically ventilated ICU patients reported that remimazolam tosylate was noninferior to propofol for short‐term sedation and was generally well tolerated [10]. Other ICU studies have reported effective sedation compared with propofol or dexmedetomidine, with potential advantages in hemodynamic stability or bradycardia risk [11, 12]. These findings support remimazolam as a feasible ICU sedative. They do not, however, settle the question of delirium. Most available trials were designed around sedation success or short‐term safety rather than postoperative delirium as the main endpoint. Our study adds real‐world data on delirium, but the evidence remains insufficient for a firm conclusion.

The longer duration of mechanical ventilation observed in the remimazolam group deserves attention. The association persisted after IPTW adjustment, but causality cannot be inferred. Patients selected for remimazolam may have required deeper or longer sedation or may have had perioperative features not fully captured in the dataset. Extubation practices, analgesic exposure, sedation depth, and readiness‐to‐wean decisions were not standardized for research purposes. In addition, although remimazolam is usually described as rapidly metabolized, drug handling in critically ill patients may be affected by inflammation, organ dysfunction, and interindividual variability. These issues make it difficult to attribute prolonged ventilation to the drug itself.

From a clinical standpoint, these findings support caution rather than a strong recommendation for or against remimazolam. Remimazolam may be useful when hemodynamic stability is a priority. At the same time, it should not be assumed to be neutral or protective with respect to delirium in postoperative ICU patients. In patients at high risk for delirium, careful titration, daily reassessment of sedation needs, and routine delirium screening remain important regardless of the sedative chosen.

## 5. Limitations

This study has several limitations. First, it was conducted at a single center, which may limit generalizability to institutions with different surgical case mix, sedation protocols, and extubation practices. Second, the number of delirium events was small, resulting in limited precision and wide confidence intervals. Third, the comparator group was heterogeneous and included patients receiving different sedative agents or no continuous sedative infusion. Fourth, residual confounding may remain despite IPTW adjustment, especially for sedation depth, extubation readiness, analgesic exposure, and physician decision‐making. Fifth, delirium assessment may have been limited during periods of deep sedation. Finally, the study did not assess long‐term cognitive outcomes after ICU discharge.

## 6. Conclusions

In this single‐center prospective cohort study, remimazolam was not statistically significantly associated with postoperative delirium compared with nonremimazolam sedation strategies. Because the number of delirium events was small and the comparator group was heterogeneous, clinically important differences cannot be excluded. Remimazolam was associated with longer mechanical ventilation duration, a finding that should be interpreted cautiously in view of possible residual confounding. Larger studies with prespecified delirium endpoints and more homogeneous comparator groups are needed.

## Author Contributions

Jiajia Hong and Chengjin Zhao contributed to data collection, data verification, and manuscript drafting. Lin Lin contributed to study conception, supervision, interpretation of findings, and critical revision of the manuscript.

## Funding

This study received no external funding.

## Disclosure

All authors approved the final version of the manuscript.

## Conflicts of Interest

The authors declare no conflicts of interest.

## Supporting Information

Additional supporting information can be found online in the Supporting Information section.

## Supporting information


**Supporting Information** Supporting Table S1. Multigroup comparisons by comparator sedation strategy. These analyses were exploratory because of small subgroup sizes and zero‐event cells. Comparisons are presented with remimazolam as the reference group. Supporting Table S2. Sensitivity analysis excluding zero‐event subgroups. These analyses were exploratory because of small subgroup sizes and zero‐event cells. Comparisons are presented with remimazolam as the reference group.

## Data Availability

The data that support the findings of this study are available from the corresponding author upon reasonable request. The data are not publicly available because of privacy and ethical restrictions.
