# Supplementary material for: Effect of Remimazolam on Postoperative Delirium in Surgical ICU Patients: A Single‐Center Prospective Cohort Study
Source: Crit Care Res Pract. 2026 Jun 18;2026:3315388. doi: 10.1155/ccrp/3315388 (PMC13277771; doi:10.1155/ccrp/3315388)
Supplement: Supplementary file 1 — Supporting Information Supporting Table S1. Multigroup comparisons by comparator sedation strategy. These analyses were exploratory because of small subgroup sizes and zero‐event cells. Comparisons are presented with remimazolam as the reference group. Supporting Table S2. Sensitivity analysis excluding zero‐event subgroups. These analyses were exploratory because of small subgroup sizes and zero‐event cells. Comparisons are presented with remimazolam as the reference group. [file CCRP-2026-3315388-s001.docx]

**Supplementary Material**

**Supplementary Table S1. Multi-group comparisons by comparator sedation strategy**

These analyses were exploratory because of small subgroup sizes and zero-event cells. Comparisons are presented with remimazolam as the reference group.

| **Outcome** | **Comparison** | **OR or median difference (95% CI)** | **P** |
| --- | --- | --- | --- |
| Delirium, n (%) | No continuous sedation vs Remimazolam | 0.22 (0.01, 4.11) | 0.308 |
| Delirium, n (%) | Dexmedetomidine vs Remimazolam | 0.91 (0.28, 2.90) | 0.869 |
| Delirium, n (%) | Midazolam vs Remimazolam | 0.25 (0.01, 4.89) | 0.362 |
| Delirium, n (%) | Propofol/ciprofol vs Remimazolam | 2.21 (0.44, 11.02) | 0.332 |
| Duration of mechanical ventilation, h | No continuous sedation vs Remimazolam | -7.00 (-9.00, -2.00) | 0.004 |
| Duration of mechanical ventilation, h | Dexmedetomidine vs Remimazolam | -6.00 (-8.00, -0.48) | 0.052 |
| Duration of mechanical ventilation, h | Midazolam vs Remimazolam | -5.00 (-7.00, -1.00) | 0.032 |
| Duration of mechanical ventilation, h | Propofol/ciprofol vs Remimazolam | -7.00 (-9.00, 5.00) | 0.260 |
| ICU length of stay, h | No continuous sedation vs Remimazolam | -1.58 (-6.00, 1.37) | 0.264 |
| ICU length of stay, h | Dexmedetomidine vs Remimazolam | 0.83 (-3.18, 18.02) | 0.688 |
| ICU length of stay, h | Midazolam vs Remimazolam | -1.80 (-5.07, -0.15) | 0.036 |
| ICU length of stay, h | Propofol/ciprofol vs Remimazolam | -0.25 (-5.00, 78.79) | 0.980 |
| Hospital length of stay, d | No continuous sedation vs Remimazolam | 0.00 (-2.00, 3.00) | 1.000 |
| Hospital length of stay, d | Dexmedetomidine vs Remimazolam | -1.00 (-3.00, 2.00) | 0.788 |
| Hospital length of stay, d | Midazolam vs Remimazolam | 0.00 (-3.00, 5.00) | 1.000 |
| Hospital length of stay, d | Propofol/ciprofol vs Remimazolam | 5.00 (-6.00, 9.00) | 0.400 |
| Relative change in CRP, % | No continuous sedation vs Remimazolam | 932.50 (-722.86, 5956.87) | 0.160 |
| Relative change in CRP, % | Dexmedetomidine vs Remimazolam | 116.91 (-416.58, 941.24) | 0.900 |
| Relative change in CRP, % | Midazolam vs Remimazolam | 2188.45 (299.65, 3654.18) | 0.004 |
| Relative change in CRP, % | Propofol/ciprofol vs Remimazolam | -814.65 (-1272.92, 807.96) | 0.124 |

**Supplementary Table S2. Sensitivity analysis excluding zero-event subgroups**

These analyses were exploratory because of small subgroup sizes and zero-event cells. Comparisons are presented with remimazolam as the reference group.

| **Outcome** | **Comparison** | **OR or median difference (95% CI)** | **P** |
| --- | --- | --- | --- |
| Delirium, n (%) | Dexmedetomidine vs Remimazolam | 0.91 (0.28, 2.90) | 0.869 |
| Delirium, n (%) | Propofol/ciprofol vs Remimazolam | 2.21 (0.44, 11.02) | 0.332 |
| Duration of mechanical ventilation, h | Dexmedetomidine vs Remimazolam | -6.00 (-8.00, -0.48) | 0.052 |
| Duration of mechanical ventilation, h | Propofol/ciprofol vs Remimazolam | -7.00 (-9.00, 5.00) | 0.312 |
| ICU length of stay, h | Dexmedetomidine vs Remimazolam | 0.83 (-4.79, 18.39) | 0.712 |
| ICU length of stay, h | Propofol/ciprofol vs Remimazolam | -0.25 (-5.00, 23.99) | 1.000 |
| Hospital length of stay, d | Dexmedetomidine vs Remimazolam | -1.00 (-3.00, 2.00) | 0.792 |
| Hospital length of stay, d | Propofol/ciprofol vs Remimazolam | 5.00 (-6.00, 8.52) | 0.432 |
| Relative change in CRP, % | Dexmedetomidine vs Remimazolam | 116.91 (-388.00, 936.64) | 0.944 |
| Relative change in CRP, % | Propofol/ciprofol vs Remimazolam | -814.65 (-1311.39, 95.81) | 0.080 |
